# Imposter Syndrome Among Medical Professionals in General Surgery: A Multi-center Observational Study

**DOI:** 10.7759/cureus.93206

**Published:** 2025-09-25

**Authors:** Kristie Kear, Ukoha Kalu, Roland Fernandes

**Affiliations:** 1 General Surgery, East Kent Hospitals University NHS Foundation Trust, Ashford, GBR

**Keywords:** cips, general surgery, imposter phenomenon, medical staff, surgeon burnout

## Abstract

Background: Imposter syndrome (IS) occurs when high-achieving individuals have a pervasive sense of self-doubt combined with fear of being exposed as a fraud, despite objective measures of success in their career. Among groups of high-achieving individuals, IS has been identified as a major contributor to burnout, endangering their mental and emotional well-being. Insufficient research has been conducted regarding the seriousness of IS among medical staff in general surgery departments. This study aimed to determine the prevalence and severity of IS among a population of medical staff working in general surgery in district general hospitals (DGHs) in the Southeast of England.

Materials and Methods: This was a cross-sectional survey design. An anonymous Microsoft form containing socio-demographic characteristics and the Clance Imposter Phenomenon Scale (CIPS) was shared with medical staff across seven DSGs who had provided informed consent to take part in the study. The data was exported to IBM SPSS version 20 and analysed using descriptive analysis.

Results: A total of 76 out of 150 medical staff responded to the survey with a response rate of 51%. Most of the respondents were male (40, 52.6%), aged 35-44 years (42, 55.3%), of Asian or Asian British ethnicity (37, 48.7%), and staff grade ST3-ST8 (33, 43.4%). The overall mean CIPS among all the respondents was 66.41±14.68, with a range of 20 to 96. The respondents demonstrated various levels of IS, with 6.6%, 25.0%, 53.3% and 13.1% reporting mild, moderate, significant and intense IS, respectively.

Conclusions: There was a high prevalence of IS among the medical staff in general surgery. There was a statistically significant difference in moderate to intense IS CIPS among the female gender and those aged 35 to 44 years. However, there was no statistically significant difference in reported CIPS among respondents' staff grade and ethnicity.

## Introduction

Clance and Imes first described imposter syndrome (IS), also known as imposter phenomenon, in 1978 as an internal experience that is characterised by a chronic sense of self-doubt, coupled with a constant worry of being discovered as a fraud. These feelings are present despite professional accomplishments and often remarkable academic achievements [1]. Several studies have identified a relatively high prevalence of IS regardless of age, gender, or ethnicity [2-5], These prior studies have not consistently found demographic associations with IS; they remain not fully studied in general surgery staff in multiple hospitals. In the surgical communities of general surgery and neurosurgery, IS has been demonstrated to be one of the many factors that increase burnout, depression, and anxiety among healthcare workers [2,6,7]. This has resulted in the acknowledgement of IS as a contributor to the burnout phenomenon, due to emotional and psychological exhaustion. Furthermore, IS has been shown to reduce workforce efficiency and effectiveness, which is linked to poor health, alcoholism, depression and sometimes suicidal ideations among physicians [4,8].

The Clance Imposter Phenomenon Scale (CIPS) is a validated survey for IS and it consists of 20 questions on a 5-point Likert scale of 1 (not at all true) to 5 (very true) that investigates self-assessment of feelings regarding competency, praise, and success (see Appendix) [9]. The CIPS ranges from 20 to 100. If the total score is ≤40, the respondent has mild imposter characteristics; if the score is between 41 and 60, the respondent has moderate imposter characteristics; a score between 61 and 80 indicates the respondent has significant imposter characteristics; and a score ≥80 means the respondent often has intense imposter characteristics. The higher the score, the more frequently and seriously IS interferes in an individual's life [8]. The CIPS has proven to have strong content validity as it has been assessed to evaluate IS efficiently [8]. Additionally, the CIPS has demonstrated construct validity, meaning that it measures the characteristics that it is designed to test in an individual who is experiencing IS [8]. During the study by El-Ashry et al., the CIPs score was able to reliably indicate those affected by IS; however, due to CIPS lacking separate sub-scales, there was no indication of whether this could be related to the participants' social demographics [3].

There are a few studies that have focused on the severity of IS among physicians, orthopaedic surgeons, neurosurgeons, and medical students [5,10]. Nevertheless, there are limited studies on the IS severity among medical staff in general surgery, despite general surgery having a large workforce among the surgical community [11]. Therefore, the primary aim of this study was to determine prevalence and severity, and the secondary aim was to explore the IS associations with socio-demographic variables, including age, gender, ethnicity, and staff grade, among the medical staff of general surgery across multiple hospitals in the southeast of England.

## Materials and methods

Study design 

This was a cross-sectional observational multicenter study involving all general surgery medical staff in seven district general hospitals (DGHs) in East Kent in southeast of England. An unlinked anonymised online questionnaire comprising social demographic characteristics and CIPS score was conducted by a convenience sampling technique on the cohort of general surgery medical staff. The survey was conducted using a Microsoft Forms survey that was sent to the respondents’ emails. Each respondent completed the online survey voluntarily and anonymously. The respondents’ consent was implied by the individual’s willingness to complete the survey. A convenience sampling technique was used for this quantitative study until the sample size for this study was reached.

Study duration

The data collection period was 16 weeks, from January 1, 2025, to April 30, 2025. The respondents had 16 weeks to complete the survey. After the first notification, two separate reminder emails were sent to all participants in Weeks 8 and 16, and the survey period was completed at the end of Week 16. The reminder emails were sent to all participants across the hospitals to reduce bias in response rate.

Survey content

The survey consisted of two parts: social demographics and the validated CIPS survey questions. Social demographics included age, gender, ethnicity and staff grade: foundation year one doctor (FY1), foundation year two doctor (FY2), CT1-CT2 (training grade), senior house officer (SHO, non-training grade), specialty doctors (SAS), registrar trainees (ST3 -ST8), post-trainee doctors (post-certificate of completion of training (CCT)) and consultants. All the respondents with CIPS ≥ 40 were considered to experience IS.

Ethical approval

The United Kingdom Health Research Authority (HRA) decision tool concluded this study to be research [12]. Therefore, the ethical clearance for the study was obtained from the Research, Ethics, and Innovation Committee (REC) of the East Kent Hospitals University NHS Trust with the study approval number 2025/GAP/25. Permission was obtained from Professor Pauline Rose Clance, who holds the copyright permission for the CIPS used for this study [1].

Statistical analysis

The discrete variables were reported as frequencies and percentages, while continuous variables were reported as the mean and standard deviation with the range. The CIPS were compared between groups using a univariate and a multivariate analysis of variance (ANOVA) and independent-sample t-tests. The collated data was analyzed using IBM SPSS Version 25. The level of significance was set at p<0.05.

## Results

Characteristics of the respondents

Table 1 shows a total of 76 general surgery medical staff working in East Kent DGH hospitals. 76 out of 150 participants responded to the survey, which represents a 51% response rate. Most of the respondents were male (40, 52.6%), aged 35-44 years (42, 55.3%), of Asian or Asian British ethnicity (37, 48.7%), and staff grade ST3-ST8 (33, 43.4%). 

**Table 1:** Demographic characteristics of respondents (n=76) SHO: Senior house officer; SAS: Specialist, associate specialist, or specialist doctor; CTT: Certificate of completion of training

Variables		Respondents n (%)
Gender	Male	40 (52.6)
	Female	33 (43.4)
	Prefer not to say	3 (4.0)
Age (years)	20-24	1 (1.3)
	25-34	23 (30.3)
	35-44	42 (55.3)
	45-54	9 (11.8)
	55-64	1 (1.3)
Ethnicity	White/Caucasian	25 (32.9)
	Asian or Asian British	37 (48.7)
	Mixed or multiple ethnic groups	4 (5.3)
	Black, Black British, Caribbean or African	1 (1.3)
	Other	7 (9.2)
	Prefer not to say	2 (2.6)
Staff grade	FY1-FY2	19 (25.0)
	CT1-CT2	6 (7.9)
	SHO	6 (7.9)
	ST3 – ST8	33 (43.4)
	Specialty doctors (SAS)	3 (4.0)
	Post-CCT Fellow	1 (1.3)
	Consultants	8 (10.5)

CIPS distribution among the respondents

All respondents with CIPS≥40 were considered to experience at least moderate IS. The CIPS score distribution among the demographic characteristics demonstrated that female gender, age of 35-44 years, Asian or Asian British ethnicity, and ST3-ST8 trainee doctors have higher levels of CIPS compared to other respondents, as demonstrated in Table 2.

**Table 2:** Analysis of CIPS with social demographic variables CIPS: Clance Imposter Phenomenon Scale; SHO: Senior house officer; SAS: Specialist, associate specialist, or specialist doctor; CTT: Certificate of completion of training

Variables		Respondents (n)	CIPS 20-40 Mild	CIPS 41-60 Moderate	CIPS 61-80 Severe	CIPS 81-100 Intense
Gender	Male	40	4	12	22	2
	Female	33	1	7	19	6
	Prefer not to say	3	0	0	0	3
Age (years)	20-24	1	0	1	0	0
	25-34	23	2	8	9	4
	35-44	42	3	8	25	6
	45-54	9	0	2	6	1
	55-64	1	0	0	1	0
Ethnicity	White/Caucasian	25	1	6	15	3
	Asian or Asian British	37	3	7	22	5
	Mixed or multiple ethnic groups	4	0	2	2	0
	Black, Black British, Caribbean, or African	1	0	1	0	0
	Other	7	1	2	2	2
	Prefer not to say	2	0	1	0	1
Staff grade	FY1-FY2	19	3	5	9	2
	CT1-CT2	6	1	2	2	1
	SHO	6	1	3	0	2
	ST3-ST8	33	0	7	22	4
	Specialty doctors (SAS)	3	0	1	2	0
	Post-CCT Fellow	1	0	0	1	0
	Consultants	8	0	1	5	2

Severity of clinical IS and its symptomatology within the respondents

A total of 71 respondents (93.4%) had moderate to intense IS among the medical staff in general surgery, with 10 (13.1%) of the respondents showing intense IS, as highlighted in Table 3.

**Table 3:** Clinical IS and its symptomatology within the respondents IS: Imposter syndrome; CIPS: Clance Imposter Phenomenon Scale

Variables		Respondents n (%)
IS symptoms score category	Mild (CIPS < 40)	5 (6.6)
	Moderate (CIPS 41-60)	19 (25.0)
	Severe (CIPS 61-80)	42 (55.3)
	Intense (CIPS > 80)	10 (13.1)

Mean CIPS analysis

The overall mean CIPS among all the respondents was 66.41±14.68 SD, with a CIPS range of 20 to 96, which denotes a significant IS. When the respondents' mean CIPS were compared, there was a statistically significant difference in gender (p=0.01) and age (p≤0.01). However, there was no statistically significant difference in the respondents based on ethnicity (p=0.61) and staff grade (p=0.41), as shown in Table 4.

**Table 4:** Analysis of IS means score of respondents One-way ANOVA test *p-value significant at <0.05 IS: Imposter syndrome; ANOVA: Analysis of variance; SHO: Senior house officer; SAS: Specialist, associate specialist, or specialist doctor; CTT: Certificate of completion of training

Variables	Variables	Respondents (n)	CIPS Mean ± SD	F-value	p-value
Gender	Male	40	62.68 ± 14.01	5.72	0.01*
	Female	33	66.94 ± 13.92		
	Prefer not to say	3	88.33 ± 2.08		
Age (years)	20-24	1	58.00 ± 0.00	5.85	<0.01*
	25-34	23	64.65 ± 15.27		
	35-44	42	66.90 ± 15.86		
	45-54	9	68.33 ± 7.29		
	55-64	1	77.00 ± 0.00		
Ethnicity	White/Caucasian	25	67.24 ± 12.96	1.02	0.61
	Asian or Asian British	37	67.49 ± 15.71		
	Mixed or multiple ethnic groups	4	61.75 ± 4.79		
	Black, Black British, Caribbean or African	1	52.00 ± 0.00		
	Other	7	60.71 ± 17.91		
	Prefer not to say	2	72.50 ± 24.75		
Staff grade	FY1-FY2	19	61.42 ± 17.31	1.07	0.41
	CT1-CT2	6	63.83 ± 21.82		
	SHO	6	62.83 ± 19.10		
	ST3-ST8	33	68.91 ± 11.82		
	Specialty doctors (SAS)	3	65.00 ± 10.82		
	Post-CCT Fellow	1	67.00 ± 0.00		
	Consultants	8	73.00 ± 10.13		

## Discussion

In this cross-sectional study of 76 medical staff working in general surgery departments across East Kent, it has been demonstrated that moderate to intense imposterism was observed in most of the respondents, with only 6.6% of the respondents showing mild IS. In various surgical subspecialties, IS is highly variable, with a previous study showing a range of 9% to 82% [3].

When comparing the respondents by ethnicity and staff grade, the level of imposterism experienced by individuals from this study was the same as demonstrated in previous studies [2,3,8,13]. Since the first publication of Clance and Imes regarding IS among the medical workforce, like our studies, more female respondents have experienced imposter-like feelings compared to the male participants [2,5]. This could be, as Clance and Imes hypothesised, due to most females failing to internalise success the same way men do, resulting in lower self-esteem and imposter feelings [1]. This has also been demonstrated in another study that found the female gender to be positively correlated with imposter feelings in medical students and internal medicine residents [14].

There was a significant positive trend between the most represented ethnic groups in this study (those from white/Caucasian and Asian/Asian British backgrounds) and feelings of imposterism. Participants from ethnic minority groups (mixed ethnicity, Black/Black British and Caribbean/African backgrounds) were found to have less severe feelings of imposterism. This contrasts with other studies [8] that show ethnic minority groups to have higher rates of IS and white/Caucasian or Asian/Asian British participants to have lower rates of IS.

Unlike earlier research by El-Ashry et al. and Bravata et al. that used a monogenous staff grade of respondents, this study included a heterogeneous population of respondents with a range of staff grades (from foundation doctors to consultants) similar to Leach et al. [3,6,8]. The findings from this study indicate that the degree of IS was similar across all staff grades, since the CIPS did not rise or fall in tandem with respondents' years of experience. This could imply that IS may not be lessened by more years of experience in the general surgery specialty. In contrast, Leach et al., whose CIPS did not achieve statistical significance, discovered that reported CIPS declined with years of practice [6].

The findings in this study revealed that a higher proportion of younger medical staff (35-44 years) presented with elevated CIPS scores, which may affect their confidence and accurate self-assessment of strengths and limitations. This dynamic could imply the presence of a likely significant psychological burden on these young trainees. IS has been linked to burnout, indicating that those who experience it may be at increased risk of work-related exhaustion [10,15]. Future studies should thus concentrate on determining how outside variables could influence the emergence of IS and developing further techniques to lessen its effects.

IS is prevalent among surgical professionals and other healthcare workers for a variety of reasons [3, 4, 7]. Despite the results from this study not showing a significant difference by grade, IS may be more severe among junior medical staff during seniors’ assessment of their performance [7, 16]. Other reasons for high levels of IS among medical staff could include the constantly evolving regulations and guidelines expected to be recalled by individuals. Furthermore, the competitive component brought about by a shortage of training posts, unequal remuneration, or the overall “exceeding expectations” culture of the medical profession could possibly be among the contributing factors to the high prevalence of IS within the workforce [6].

Dealing with the negative emotions of IS requires acknowledgement that most people experience it at some point in their lives [15-17]. For some professionals, IS symptoms might help them advance in their careers, but for others, these feelings can lead to problems with their mental health. As a result, ongoing introspection by clinical and educational supervisors to improve the self-esteem of trainees in surgical specialties is crucial in mitigating the worrisome effects of imposterism [3]. Taking the appropriate steps to overcome these imposter feelings will help junior medical staff to progress in their surgical professions. Some studies have suggested social support, validation of success, positive affirmation, and institutional support to help to alleviate IS [4,7,8]. Additional research conducted on medical students has indicated that individual changes such as documenting achievements, acknowledging achievements, and soliciting the guidance of mentorship may alleviate feelings of IS [6,8,11].

The phenomenon of burnout may be characterised by a high degree of emotional weariness and depersonalisation, as well as a poor sense of personal accomplishment. IS and burnout have been shown to be related in some research [3,6]. However, by focusing on the higher staff grade (consultants), this study is unable to show the extent of burnout among the respondents. As has been shown in earlier studies, future studies should explore how IS contributes to emotional fatigue, skepticism, care for patients, and job satisfaction among the general surgery medical staff [2,4,7].

Limitations of the study

A greater response rate is necessary to show that the findings of this study are generalisable, as the comparatively limited number of replies from such a cohort would have impacted the sub-analysis of the results. This could be due to the two reminders across the 16-week period potentially causing lower response rates to the survey. The convenience sampling technique used in this study could risk selection bias and limits the generalisability of the results from this study.

Selection bias should also be considered a limitation, as it could be the case that clinicians with IS characteristics could be more inclined to complete the survey than more self-assured medical staff. Furthermore, the relationship between IS and burnout, personality, self-esteem, emotional tiredness, and patient care was not shown in this study. Participants of this study were not questioned regarding how IS impacts their clinical performance; however, this would be important to consider in future research.

## Conclusions

This study highlights the prevalence of IS among medical professionals working in general surgery, with most participants experiencing moderate to intense levels of self-doubt despite their qualifications and achievements. The findings underscore that IS is not confined to early-career professionals but affects individuals across various staff grades, with significant associations observed in female respondents and respondents in the middle-aged group.

These findings from this study point to the need for targeted strategies and support systems within surgical departments to address the psychological burdens associated with IS. Further research is needed to explore the causes, consequences, and effective interventions to mitigate the impact of IS. We recommend institutional interventions, mentoring and support programs to mitigate the impacts of IS among the medical staff in surgical and broader medical environments.

## Appendices

**Table 5:** CIPS and social demographic questionnaire Credit to Professor Pauline Rose Clance for the usage of CIPS questionnaire. CIPS: Clance Imposter Phenomenon Scale; SHO: Senior house officer; SAS: Specialist, associate specialist, or specialist doctor; CTT: Certificate of completion of training

For each question, please select the number that best indicates how true the statement is of you.					
Question	1. Not at all true	2. Rarely	3. Sometimes	4. Often	5. Very true/the majority of the time
I have often succeeded on a test or task even though I was afraid that I would not do well before I undertook the task.					
I can give the impression that I’m more competent than I really am.					
I avoid evaluations if possible and have a dread of others evaluating me.					
When people praise me for something I’ve accomplished, I’m afraid I won’t be able to live up to their expectations of me in the future.					
I sometimes think I obtained my present position or gained my present success because I happened to be in the right place at the right time or knew the right people.					
I’m afraid people important to me may find out that I’m not as capable as they think I am.					
I tend to remember the incidents in which I have not done my best more than those times I have done my best.					
I rarely do a project or task as well as I’d like to do it.					
Sometimes I feel or believe that my success in my life or in my job has been the result of some kind of error.					
It’s hard for me to accept compliments or praise about my intelligence or accomplishments.					
At times, I feel my success has been due to some kind of luck.					
I’m disappointed at times in my present accomplishments and think I should have accomplished much more.					
Sometimes I’m afraid others will discover how much knowledge or ability I really lack.					
I’m often afraid that I may fail at a new assignment or undertaking even though I generally do well at what I attempt.					
When I’ve succeeded at something and received recognition for my accomplishments, I have doubts that I can keep repeating that success.					
If I receive a great deal of praise and recognition for something I’ve accomplished, I tend to discount the importance of what I’ve done.					
I often compare my ability to those around me and think they may be more intelligent than I am.					
I often worry about not succeeding with a project or examination, even though others around me have considerable confidence that I will do well.					
If I’m going to receive a promotion or gain recognition of some kind, I hesitate to tell others until it is an accomplished fact					
I feel bad and discouraged if I’m not “the best” or at least “very special” in situations that involve achievement.					
Social Demographic Questions					
What is your grade/position?	☐ FY1 ☐ FY2 ☐ SHO ☐ CT1 ☐ CT2 ☐ ST3 ☐ ST4 ☐ ST5 ☐ ST6 ☐ ST7 ☐ ST8 ☐ SAS ☐ Post-CCT Fellow ☐ Consultant				
What is your gender identity?	☐ Female ☐ Male ☐ Transgender ☐ Nonbinary ☐ Other ☐ Prefer not to say				
What age category do you fall into?	☐ 20-24 years old ☐ 25-34 years old ☐ 35-44 years old ☐ 45-54 years old ☐ 55-64 years old ☐ 65+ years old				
What is your ethnic background?	☐ White/Caucasian ☐ Asian or Asian British ☐ Black, Black British, Carribbean or African ☐ Mixed or multiple ethnic groups ☐ Other ☐ Prefer not to say				
